# Investigation of the Effect of Nanocrystalline Calcium Carbonate-Substituted Hydroxyapatite and L-Lysine and L-Arginine Surface Interactions on the Molecular Properties of Dental Biomimetic Composites

**DOI:** 10.3390/biomimetics6040070

**Published:** 2021-12-10

**Authors:** Dmitry Goloshchapov, Vladimir Kashkarov, Kirill Nikitkov, Pavel Seredin

**Affiliations:** 1 Solid State Physics and Nanostructures Department, Voronezh State University, University Sq. 1, 394018 Voronezh, Russia; goloshchapovdl@gmail.com (D.G.); vmkashkarov@gmail.com (V.K.); nikitkov.vsu@gmail.com (K.N.); 2 Scientific and Educational Center “Nanomaterials and Nanotechnologies”, Ural Federal, Mir Av., 620002 Yekaterinburg, Russia

**Keywords:** biocompatible materials, biomimetics, nanocrystalline carbonate-substituted hydroxyapatite, L-Lysine, L-Arginine, macromolecular substances, molecular properties, interaction

## Abstract

Differences in the surface interactions of non-stoichiometric nanocrystalline B-type carbonate-substituted hydroxyapatite (n-cHAp) with the amino acids L-Lysine hydrochloride (L-LysHCl) and L-Arginine hydrochloride (L-ArgHCl) in acidic and alkaline media were determined using structural and spectroscopic analysis methods. The obtained data confirm that hydroxyapatite synthesized using our technique, which was used to develop the n-cHAp/L-LysHCl and n-cHAp/L-ArgHCl composites, is nanocrystalline. Studies of molecular composition of the samples by Fourier transform infrared spectroscopy under the change in the charge state of L-Lysine in environments with different alkalinity are consistent with the results of X-ray diffraction analysis, as evidenced by the redistribution of the modes’ intensities in the spectra that is correlated with the side chains, i.e., amide and carboxyl groups, of the amino acid. During the formation of a biomimetic composite containing L-Lysine hydrochloride and n-cHAp, the interaction occurred through bonding of the L-Lysine side chain and the hydroxyl groups of hydroxyapatite, which created an anionic form of L-Lysine at pH ≤ 5. In contrast, in biocomposites based on L-Arginine and n-cHAp, the interaction only slightly depends on pH value, and it proceeds by molecular orientation mechanisms. The X-ray diffraction and infrared spectroscopy results confirm that changes in the molecular composition of n-cHAp/L-ArgHCl biomimetic composites are caused by the electrostatic interaction between the L-ArgHCl molecule and the carbonate-substituted calcium hydroxyapatite. In this case, the bond formation was detected by Fourier transform infrared (FTIR) spectroscopy; the vibrational modes attributed to the main carbon chain and the guanidine group of L-Arginine are shifted during the interaction. The discovered interaction mechanisms between nanocrystalline carbonate-substituted hydroxyapatite that has physicochemical properties characteristic of the apatite in human dental enamel and specific amino acids are important for selecting the formation conditions of biomimetic composites and their integration with the natural dental tissue.

## 1. Introduction

Biomimetic composites containing an organic matrix and inorganic phosphate components are promising materials for restorative and regenerative dentistry, surgery and bone grafting [1,2,3]. The synthesis of such hybrid biomaterials, reproducing the physicochemical properties of human hard tissues [4,5,6,7,8], as well as the modification of biomaterials with different inorganic bioactive ions and molecular groups in order to initiate the controlled reaction in tissues and to provide antimicrobic activity, is a complex and multilevel task [9,10,11,12].

Within the framework of the biomimetic concept [13,14], reproduction of the inorganic base of tooth enamel and dentin can be best achieved by using nanocrystalline carbonate-substituted hydroxyapatite, n-cHAp, (Ca_10_(PO_4_)_6−x_(CO_3_)_x_(OH)_2−x_, 0.1 < x < 0.3), which is characterized by an excellent appropriateness with the properties of dental matrix apatite [1,15]. At the same time, a large amount of research considering the problem of dental tissue repair [5,16,17,18,19] has established the importance of using a combination of n-cHAp and polar amino acids in dental biocomposites. As subunits of the protein matrix of teeth, amino acids perform many functions, including forming a biomimetic composite with the necessary configuration to compensate for surface and volume defects in enamel and dentition. Key factors in the formation of a stable bond between the organic (protein) matrix and the phosphate complexes are not only the presence of amino acid side chains, but also the presence of uncompensated charge on the surface of the mineral complexes [20,21,22] and the conditions chosen for the synthesis processes: solvent, temperature, and pH value [23,24].

A large number of studies on the synthesis of composite materials have used amino acids in an anionic form, that is, at pH values above the isoelectric point (pH > 8) [17,18,23,25,26]. However, current enamel and dentin pre-treatment therapeutical procedures (the total-etch technique of tooth surface for chemically adhesive composite restorations), use strong acid-based solutions (pH ≤ 2) [27]. This environment may lead to a different interaction mechanism not only between the components of the biomimetic composite, but also with the natural tissue being restored. 

Thus, the issue of the mechanism of the hydroxyapatite–amino acid bond formation remains an important one, in addition to the determination of the molecular functional groups responsible for the processes of crystallisation [19,26,28].

It should be noted that using various biomedical strategies, such as a direct mineralization from a solution [14], protein/peptide induction of mineralization [29,30], and assembly with the use of the ready subunits and precursors [10,31,32], no appropriate biomimetic technologies for the restoration of the hard dental tissue for its clinical application have yet been developed (similar to the cutaneous covering) [33,34,35]; this means that the problem of the imitation of the natural interaction between organic-mineral components in the synthesized materials still remains open-ended.

Therefore, this study aimed to obtain biomimetic materials reproducing the properties of the natural hard dental tissue of a human based on nanocrystalline hydroxyapatite and amino acids (L-Lysine and L-Arginine) by changing their charge state, and, for the first time, to perform a comprehensive study of the regularities and the effects of the surface interaction mechanisms on the molecular properties of these biomimetic composites using structural and spectroscopic methods.

## 2. Materials and Methods

### 2.1. Methodology for Obtaining Samples

The formation of the biomimetic composites was conducted in an aqueous medium and occurred in several stages with the preparation of solutions containing the inorganic and organic components. Aqueous solutions were used because hydroxyapatite at the chosen synthesis conditions contains a hydrated layer through which the ion-exchange reactions occur [36]. Chemical precipitation using a biogenic calcium source according to the method described in [37] was used to obtain n-cHAp that reproduced the features of the enamel apatite. The crystal structure of the n-cHAp samples synthesized in our work is characterized by the substitution of the phosphate ion PO_4_^3−^ by CO_3_^2−^ group (B-type of substitution) in hydroxyapatite crystal lattice.

First, a homogeneous suspension containing n-cHAp was prepared. This approach resulted in a solution containing hydroxyapatite nanocrystals with a high specific surface area [37]. After conditioning the solution for 24 h to form a homogenous solution; this solution containing n-cHAp was subjected to ultrasonic stirring for prevention of the agglomeration of crystals. A Sonica Q55 55W ultrasonic homogeniser was used for this purpose, operating at the amplitude of 50 for 5 min. Concentration of the n-cHAp in homogeneous suspension was at most 18.8 mg/mL.

Aqueous solutions of amino acids were prepared using L-Lysine hydrochloride (L-LysHCl) and L-Arginine hydrochloride (L-ArgHCl) powders. The concentration of the amino acids was at most 10 mg/mL. These amino acids were dissolved in ultrapure water (Millipore Milli-Q gradient ultrapure water system, Q-Guard 1, QGARDOOR1, with 0.22 µm Millipore MILLIPAK Express 0575, MPGP02001) and sonicated (Q55 Sonica 55W) at a 50% amplitude for 5 min. The choice of the hydrochloride forms of amino acids is because polar amino acids are bound to molecules of various mineral complexes and ions in many processes, and these complexes can mediate their interaction with biogenic hydroxyapatite.

To study the changes in the charge state of the amino acids, aqueous solutions of L-LysHCl and L-ArgHCl with different pH values were prepared. For this purpose, aqueous solutions of ammonia (NH_4_OH) and hydrochloric acid (HCl) were added to the aqueous solutions of amino acids to obtain solutions of pH ≥ 11.2, ≥ 7.5 and ≤ 5. pH values were measured with an Orion 420A pH meter, and the electrode was cleaned with water before and after each measurement. For the calibration, pH-buffer solutions were used. 

In the last step, thus prepared aqueous solutions of n-cHAp and L-Lysine or L-Arginine were mechanically mixed to form a homogeneous suspension and subjected to ultrasonic agitation (Q55 Sonica 55W) at the amplitude of 50% for 10 s. The concentration of the n-cHAp to amino acids in the final solutions attained approximately 2:1. The resulting samples were dried for 24 h at 20 °C. As a result, n-cHAp/L-LysHCl and n-cHAp/L-ArgHCl biocomposites were crystallised with different pH levels.

### 2.2. Methods of Structural and Spectroscopic Analysis of the Samples

#### 2.2.1. X-ray Structural Analysis

Phase composition of all the samples was investigated using a DRON-4-07 X-ray diffractometer. The X-ray source was an X-ray tube with a cobalt anode (λ = 1.7902 Å) at the voltage of 26 kV and current of 15 mA. Phase analysis was performed using the JCPDS-ICDD database.

#### 2.2.2. Optical Microscopy

Optical images of the samples in the light field were obtained at 1000× magnification using a CX41 Olympus optical microscope.

#### 2.2.3. Transmission Electron Microscopy (TEM)

The TEM technique was used for visualisation of n-cHAp nanocrystals. The study was carried out with the electron microscope Libra 120 Carl Zeiss.

#### 2.2.4. Fourier Transform Infrared (FTIR) Spectroscopy

FTIR absorption spectra of the composites in the range 4500–400 cm^−1^. and spectral resolution of 2 cm^−1^ were recorded at the Infrared Microspectroscopy (IRM) beamline (Australian synchrotron, Clayton, VIC, Australia) using a Bruker Vertex 80v spectrometer coupled with a Hyperion 2000 FTIR microscope and a liquid nitrogen-cooled narrow-band mercury cadmium telluride (MCT) detector (Bruker Optik, Germany) [38,39,40]. Blackman-Harris 3-term apodization, Mertz phase correction, and a zero-filling factor of 2 were set as default acquisition parameters using the OPUS 7.2 software suite (Bruker Optik, Germany).

#### 2.2.5. Information about Diagnostic Methods Used in Work, Provided for Comparison

Comparative information about structural and spectroscopic methods of analysis used in our work is presented in Table 1.

## 3. Results

When the pH factor of the medium changes, the conformational environment of the amino acids is subjected to changes, which may affect the molecular structure of the biomimetic composite [16,41,42]. Therefore, the structural and molecular properties of the amino acids L-LysHCl and L-ArgHCl crystallised from solutions with different pH values were compared with the properties of n-cHAp/L-LysHCl and n-cHAp/L-ArgHCl biomimetic composites obtained under the same conditions and pH values, in order to establish the influence of the charge state of amino acids on the final biocomposite.

### 3.1. X-ray Diffractometry

**Figure 1:**
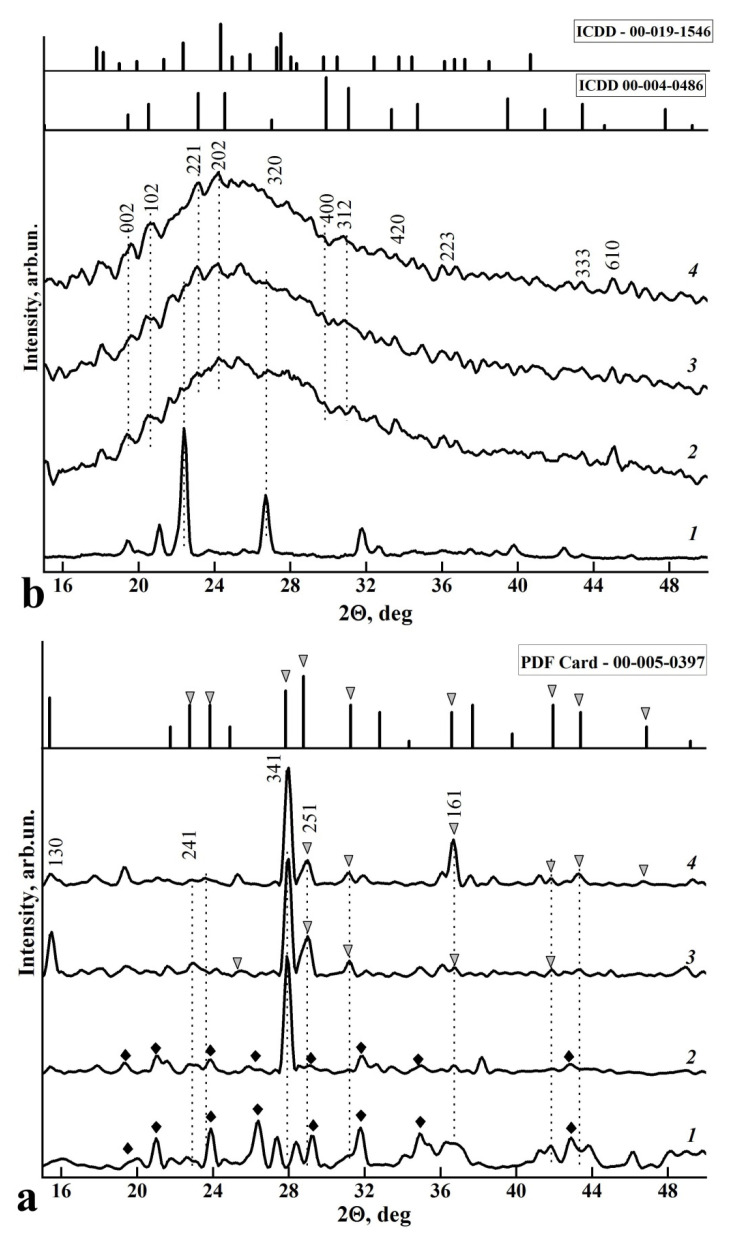
XRD scans of amino acid samples: (**a**) L-LysHCl in the original crystalline state (curve 1) and crystallised from solutions at pH ≤ 5 (curve 2), pH ≥ 7.5 (curve 3) and pH ≥ 11.2 (curve 4). (**b**) L-ArgHCl in the original crystalline state (curve 1) and crystallised from solutions with pH ≤ 5 (curve 2), pH ≥ 7.5 (curve 3) and pH ≥ 11.2 (curve 4). ▼—L-Lys diffraction reflexes, ♦—L-LysHCl diffraction reflexes. ICDD database data: L-Lysine–ICDD Card 00-005-0397; L-Arginine–ICDD Card 00-004-0486 and 00-019-1546.

Figure 1 shows the X-ray diffraction analysis (XRD) scans of amino acids L-LysHCl and L-ArgHCl before dissolution (in powder form) and crystallised from solutions with different pH values: ≥ 11.2, ≥ 7.5 and ≤ 5.

The X-ray diffraction results (Figure 1) demonstrate heterogeneous behaviour of L-Lysine and L-Arginine after crystallisation from a neutral medium (pH ≥ 7.55). The samples of L-Lysine crystallised from solution are characterised by the appearance of texture; that is, the dissolution and subsequent crystallisation of the L-Lysine changes the direction of the preferential orientation of the amino acid crystals (Figure 1a, curves 2, 3, 4). This change is represented in the increased intensity in the region of 2θ = 27.9°. At the same time, the intensity of other diffraction lines decreases proportionally. As has been repeatedly shown [18,43], the dissolution of amino acids in an aqueous medium and their subsequent crystallisation changes the conformational environment of the amino acid molecule. Therefore, peaks appear in the region of 2θ = 27.9°, 29.0°, 36.7° and 43.6° that were not observed in the diffraction spectrum of the original hydrochloride form of L-Lysine (Figure 1a). This analysis shows that a change in the charge state of the amino acid (above and below the isoelectric point of pH = 8.7) is observed as a redistribution of the intensity of the main diffraction reflexes. With increasing pH of the medium from which L-Lysine was crystallised, X-ray lines corresponding to pure L-Lysine (sample PDF Card-00-005-0397) increase their intensity (Figure 1a).

In contrast, the samples of L-Arginine crystallised from solutions are characterised by the appearance of amorphous phases. Figure 1b shows the diffractograms of powdered L-ArgHCl (curve 1) and the samples obtained following the dissolution and recrystallisation of L-ArgHCl in media with different pH values: ≥11.2, ≥7.5 and ≤5 (curves 2, 3, 4). In the background of the wide halo at 2θ = 15–50°, low-intensity reflexes near 2θ = 19.5°, 20.7°, 23.2°, 24.3°, 29.7°, 30.9° and 43.3° are observed, which are in agreement with the L-Arginine phase (ICDD 00-004-0486 and 00-019-1546). The width and intensity of the detected lines and their agreement with the ICDD data indicate a distortion in the conformational environment of L-Arginine hydrochloride and the presence of disordered amorphous phase in all samples after their crystallisation (Figure 1b).

**Figure 2:**
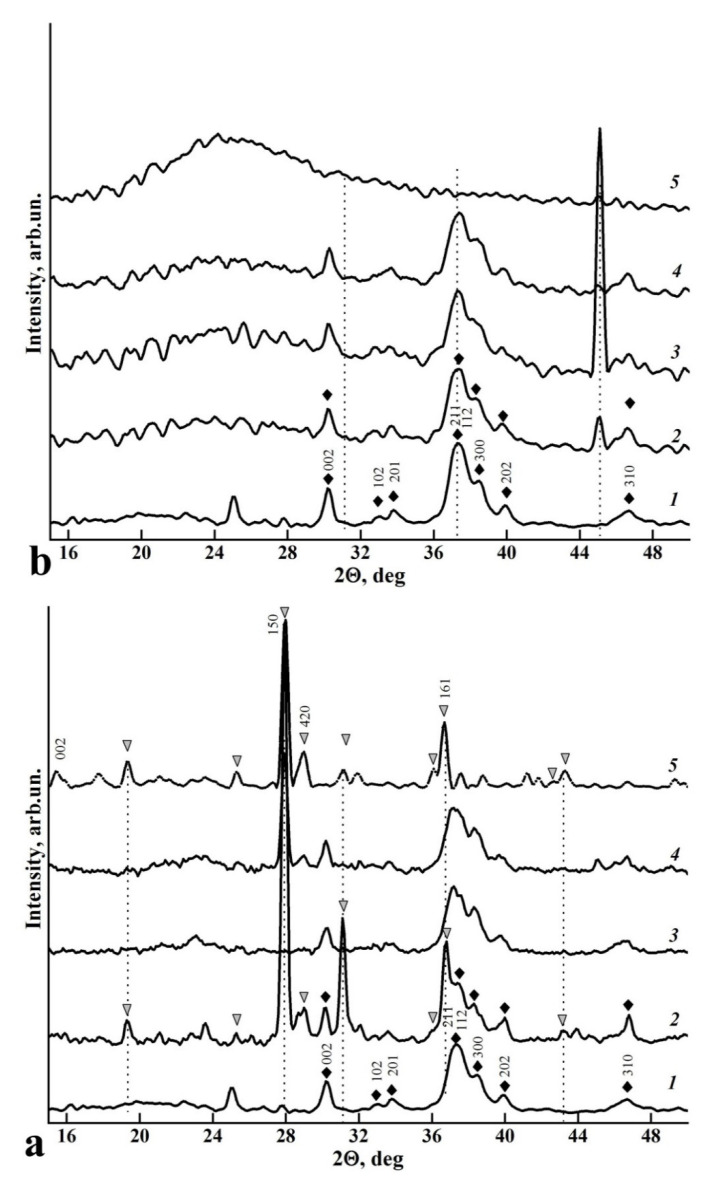
XRD scans of the biocomposite samples: (**a**) n-cHAp (curve 1); n-cHAp/L-LysHCl biocomposites obtained from solutions of pH ≤ 5 (curve 2), pH ≥ 7.5 (curve 3) and pH ≥ 11.2 (curve 4); L-LysHCl amino acid sample obtained by crystallisation from solution with pH ≥ 11.2 (curve 5). (**b**) n-cHAp (curve 1); n-cHAp/L-ArgHCl biocomposites obtained from solutions with pH < 5 (curve 2), pH ≥ 7.5 (curve 3), and pH ≥ 11.2 (curve 4); L-ArgHCl amino acid sample obtained by crystallisation from a solution with pH ≥ 11.2 (curve 5). ▼—diffraction reflexes of L-LysHCl, ♦—diffraction reflexes of n-cHAp.

The diffractograms of the n-cHAp/L-LysHCl and n-cHAp/L-ArgHCl biocomposites containing nanocrystalline B-type substituted hydroxyapatite and the amino acids L-Lysine and L-Arginine are shown in Figure 2a and Figure 2b, respectively.

The X-ray diffraction data of n-cHAp/L-LysHCl and n-cHAp/L-ArgHCl samples show that all diffraction reflections belong to calcium hydroxyapatite-substituted carbonate (ICDD 01-074-0565), L-Lysine hydrochloride (PDF Card 00-005-0397) or L-Arginine hydrochloride (PDF Card 00-004-0486 and PDF Card 00-019-1546), respectively. There is a redistribution of X-ray reflection intensities relative to ICDD data. A detailed examination of the XRD data of these biocomposites indicates the changes in the conformational environment of the amino acids in the presence of n-cHAp hydroxyapatite, distinct from those ones observed for the samples of the corresponding amino acids crystallised after dissolution at the same pH values (Figure 2). Moreover, the results show that L-Arginine hydrochloride retains its amorphous structure in the presence of hydroxyapatite.

Thus, for the n-cHAp/L-LysHCl composites crystallised from solutions with pH < 5 (Figure 2a, curve 2), an increase in the intensity of diffraction lines 2θ = 19.3°, 25.3°, 31.2°, 36.0°, 36.7°, 43.3° and 46.7° was observed. A similar ratio of intensities of the mentioned reflexes was observed for the L-LysHCl sample crystallised from the solution at pH > 11.2 (Figure 2a, curve 5). Comparison of the X-ray diffraction data shows that the distribution of X-ray diffraction intensities for the n-cHAp/L-LysHCl composite crystallised from a solution with pH > 11.2 corresponds to the L-LysHCl sample obtained from solutions with pH < 5 and pH = 7.5 (Figure 2a). 

Regarding the hydroxyapatite included in the biocomposites (Figure 2a, curve 1), a redistribution of intensity of the main diffraction reflexes of n-cHAp (112), (202) and (002) was observed for the samples n-cHAp/L-LysHCl (Figure 2a, curve 2, 3, 4). The intensity of the (112) and (202) reflex is reduced relative to the intensity of reflex (002), which means a possible occurrence of the texture—the directional agglomeration of hydroxyapatite in the sample (Figure 2a).

### 3.2. Microscopy

**Figure 3:**
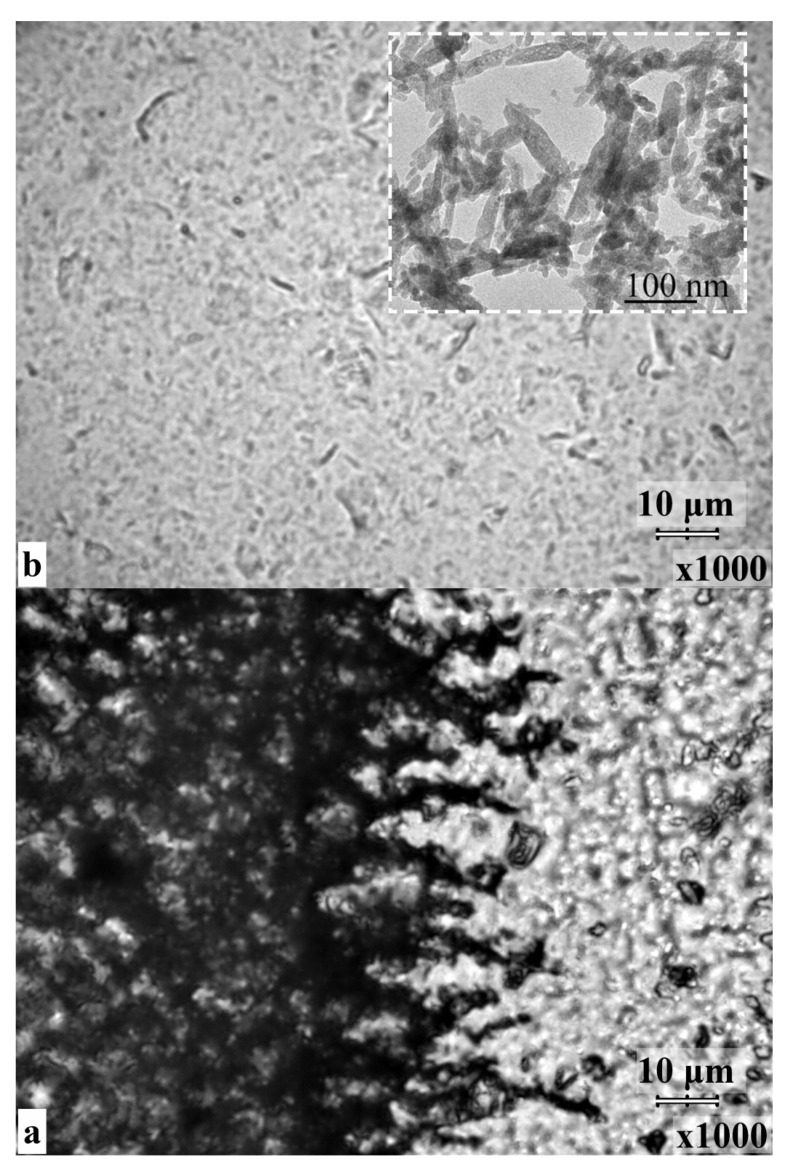
Optical images of the surface for the biocomposites (1000× magnification) (**a**) n-cHAp/L-LysHCl (pH ≥ 11.2); (**b**) n-cHAp/L-ArgHCl (pH ≥ 11.2). In the inset, the TEM image of n-cHAP nanocrystals used for the preparation of the composite is presented.

Given a high content of nanocrystalline hydroxyapatite in the amino acid complexes of the samples, the resulting biocomposites were examined using light field optical microscopy. Figure 3 shows images of the surface morphology of the n-cHAp/L-LysHCl and n-cHAp/L-ArgHCl composites (1000× magnification). The inset in Figure 3 represents the TEM image of n-cHAp nanocrystals, obtained in the work.

From the optical images of the n-cHAp/L-LysHCl sample (Figure 3a), the aggregation of hydroxyapatite particles in the biocomposite occurred on the surfaces of all the samples. Thus, the formation of large conjugates with the approximate dimensions 2 × 20 µm, oriented along a particular direction, is quite characteristic (Figure 3a). All of the particles and agglomerates of n-cHAp/L-LysHCl sample involve n-cHAp nanocrystals shown in the inset of Figure 3. In comparison, the n-cHAp/L-ArgHCl biocomposite surface is characterised by the presence of only a few large particles (Figure 3b) among a homogeneous suspension, with the main mass being ~1µm smaller agglomerates. The single large agglomerates were predominantly of the spherical or oval shape and 2–15 µm in diameter.

### 3.3. FTIR Spectroscopy

A number of studies on calcium hydroxyapatite and amino acid-based biocomposites have repeatedly demonstrated the effectiveness of Fourier transform infrared spectroscopy for analysing changes in the molecular composition of the samples [18,41,44]. In addition, as it is noted in [43,45,46,47], the activity of amino acid side chains can be determined from their vibrational spectra and they represent the conformational changes of molecules in different environments. Therefore, the use of FTIR spectroscopy, sensitive to molecular transformations, is to study the mechanisms of conjugation of proteins and mineral components in biocomposites to be optimal [40,48,49].

**Figure 4:**
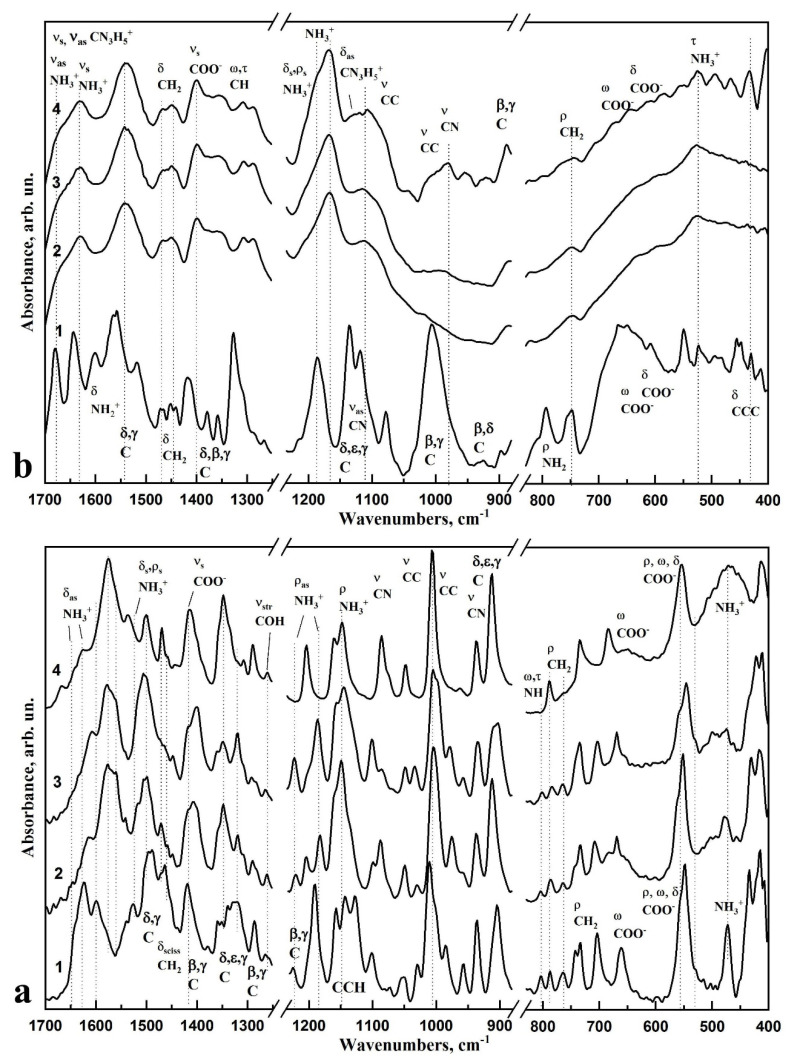
FTIR absorption spectra of the amino acid samples: (**a**) L-LysHCl in the original crystalline state (curve 1) and crystallised from solutions at pH ≤ 5 (curve 2), pH ≥ 7.5 (curve 3) and pH ≥ 11.2 (curve 4); (**b**) L-ArgHCl in the original crystalline state (curve 1) and crystallised from solutions at pH ≤ 5 (curve 2), pH ≥ 7.5 (curve 3) and pH ≥ 11.2 (curve 4).

**Figure 5:**
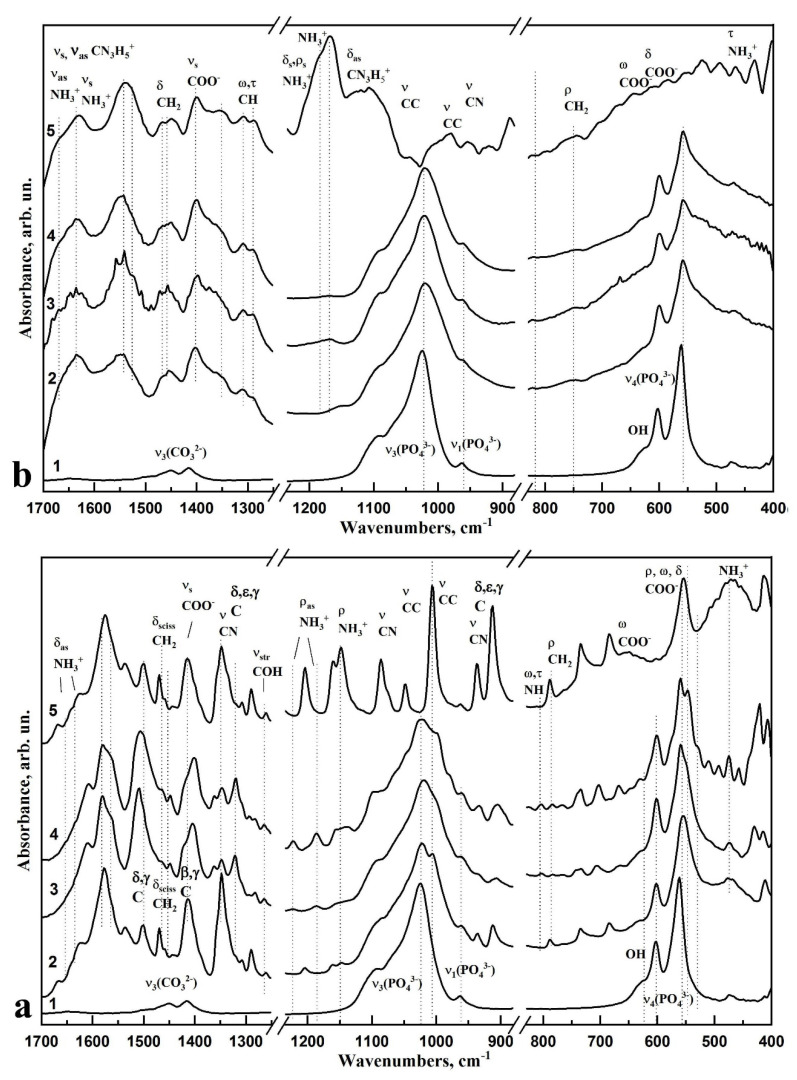
FTIR absorption spectra of biocomposite samples: (**a**) nanocrystalline B-type carbonate-substituted hydroxyapatite n-cHAp (curve 1), n-cHAp/L-LysHCl biocomposites obtained from solutions of pH ≤ 5 (curve 2), pH ≥ 7.5 (curve 3) and pH ≥ 11.2 (curve 4), L-LysHCl amino acid sample obtained by crystallisation from solution with pH ≥ 11.2 (curve 5); (**b**) nanocrystalline B-type carbonate-substituted hydroxyapatite n-cHAp (curve 1), n-cHAp/L-ArgHCl biocomposites obtained from solutions of pH ≤ 5 (curve 2), pH ≥ 7.5 (curve 3) and pH ≥ 11.2 (curve 4), L-ArgHCl amino acid sample obtained by crystallisation from solution with pH ≥ 11.2 (curve 5).

Figure 4 and Figure 5 show FTIR spectra of L-LysHCl and L-ArgHCl crystallised from solutions with different pH values, and the spectra of the n-cHAp/L-LysHCl and n-cHAp/L-ArgHCl biocomposites containing nanocrystalline B-type substituted hydroxyapatite obtained under similar conditions. In addition, the figures show the FTIR absorption spectra of the amino acids L-LysHCl and L-ArgHCl in their original crystalline state. The active modes in the FTIR spectra of the respective samples are listed in Table 2 and Table 3.

The FTIR spectra of the L-LysHCl samples (Figure 4a) show that, relative to the neutral pH ≥ 7.5, the increase and decrease of the hydrogen potential led to a redistribution of the intensity of the characteristic vibrational bands of different L-Lysine functional groups. This change reflects the variation of the charge state of the amino acid. According to the current interpretation, the NH_3_^+^ and COO^−^ side chains of amino acids should show the most prominent changes [16,41,50]. The intensities of the modes localised around 1637–1610 cm^−1^ and 1220, 1182, 805 cm^−1^, which are related to the amide (NH_3_) groups of L-Lysine, were noticeably redistributed depending on the pH value. In the case of increased alkalinity (Figure 4a, curve 3,4), the COO^−^ group vibration at 1415 cm^−1^ increased its intensity. Likewise, in an acidic medium, there was a slight redistribution of intensity of the 1530 cm^−1^ modes attributed to vibrations of the amide group (Figure 4a, curve 2). The bands in the spectrum corresponding to the rocking vibrations of the amide group (ρ, NH_3_^+^) at 1186 cm^−1^ and 1220 cm^−1^ strongly decrease up to background intensity in the case of the sample that crystallised from solutions at pH ≥ 11.2 (Figure 4a, curve 4). The spectral feature in the form of a shoulder near 1470–1460 cm^−1^ attributed to the strain vibrations of CH_2_ groups became moderately intense. In the range of 1010–930 cm^−1^, there was also a slight redistribution of intensities of the vibrations attributed to C-C bonds.

In addition, in the range of 780–700 cm^−1^, a redistribution of intensity of the modes correlated to the vibrations of the carboxyl (CH_2_) groups of L-Lysine was observed. The intensities of the modes localised around 700–600 cm^−1^ and 560, 550 cm^−1^ and related to the COO^−^ groups of L-Lysine were redistributed depending on the pH value.

FTIR spectroscopic investigation of the molecular composition of L-ArgHCl samples (Figure 4b) revealed slight changes in the vibrations of CH_2_ and CH_3_ groups (1466 cm^−1^, 1449 cm^−1^, respectively), as well as of the amide bonds at 1204, 1188, and 1167 cm^−1^ that mostly reflect the modification of the arginine chain molecules in media with changing acidity.

The FTIR spectra of n-cHAp/L-LysHCl and n-cHAp/L-ArgHCl biocomposites containing nanocrystalline B-type substituted hydroxyapatite are shown in Figure 5a and Figure 5b, respectively. In addition, the figures show the absorption spectra of n-cHAp nanocrystalline B-type substituted hydroxyapatite synthesised in this study, and the amino acids L-LysHCl and L-ArgHCl that were crystallised from solutions at pH ≥ 11.2.

In the infrared spectra of n-cHAp/L-LysHCl samples, redistribution of the intensity of the *v*_4_ and *v*_3_ modes of the phosphate group PO_4_^3−^ (602 cm^−1^, 558 cm^−1^, 1091 cm^−1^, and 1024 cm^−1^, respectively), and a decrease in the intensity of the shoulder at 630 cm^−1^ correlated with the OH group of cHAp, are observed depending on the pH of the environment and the charge state of the amino acid (Figure 5a, curves 2, 3, 4). An intensity redistribution and frequency shift of the bands attributed to the vibrations of NH_3_^+^ and COO^−^ side chains of L-Lysine around 1645–1609 cm^−1^, 1220–1148 cm^−1^ and 1415, 560–550 cm^−1^, respectively, were observed simultaneously with changes in the hydroxyapatite modes in the FTIR spectra of the biocomposites obtained in acid media (Figure 5a, curve 2). 

A detailed analysis of the FTIR spectra of the n-cHAp/L-ArgHCl composite (Figure 5b) showed that vibrations of the phosphate group PO_4_ (1027 cm^−1^, valence and strain vibrations of P=O and P-O bonds) and modes of the CO_3_ carbonate anion (1420 and 1450 cm^−1^) of hydroxyapatite were present in the range of 1700–400 cm^−1^. Nano-cHAp obtained in our work by liquid-phase synthesis has B-type structural substitution (carbonate anion CO_3_^2−^ included in the position of the PO_4_ group) [37]. After adding the amino acid to n-cHAp, bands in the range of 1470–1450 cm^−1^ were observed in the spectra of the biocomposites, which correspond to the vibrations of the C-H bond in the methylene groups of organic components. The vibrational band at 1400 cm^−1^ should be related with the total vibrations of the COO^−^ in the L-Arginine molecule, which is in agreement with the literature [41]. The band at 1640–1600 cm^−1^ should be attributed to the strain vibrations of N-H bonds in amines and NH_3_^+^ ions, as well as the symmetric and asymmetric strain vibrations of C-N-H bonds in the CN_3_H_5_^+^ group. The bands recorded at 1469 cm^−1^ and 1460 cm^−1^ correspond to the strain vibrations of C-H bonds in the structural fragments of -CH-H^−^ and -CH_2_^−^.

The appearance of a certain aggregation of n-cHAp crystals in the amino acid matrix L-ArgHCl, previously detected by X-ray diffractometry, was observed in the changes of the intensity in the absorption bands of the PO_4_ phosphate-oxygen group at 1088 cm^−1^ and 962 cm^−1^ (ν_3_ bending and ν_1_ stretching, respectively). In addition, significant changes occurred in the intensity of the modes around 1220 cm^−1^ and 1185 cm^−1^ assigned to the bending and rocking vibrations of N-H bonds in NH_3_^+^ L-Arginine ions in the media with different pH. 

The results obtained by FTIR spectroscopy are in agreement with the X-ray diffraction analysis. These findings indicate the electrostatic interaction between the L-ArgHCl molecule and the carbonate-substituted calcium hydroxyapatite n-cHAp.

## 4. Discussion

In order to restore dental enamel, a number of various materials have been developed, such as composite resins and ceramics [60,61]; however, their application could not result in the achievement of a stable restoration due to imperfections in the combination of the utilized materials and the native dental tissue [12,62]. Therefore, a tendency to reproduce the native structure of the natural hard dental tissue [13,14,62] with the use of biomimetic composites involving nanocrystalline carbonate-substituted hydroxyapatite in its composition is in the basis of the doctrine of current therapeutic dentistry.

Improving the integration of biocomposites with natural dental tissue and regenerating tissue using a biomimetic strategy requires an understanding of the mechanisms of interaction between the artificial and natural materials in the nano- and microscales [40,63,64]. The results of a comparative analysis of the data obtained by a set of structural and spectroscopic diagnostic methods reveal the differences in the mechanisms of amino acid–nanocrystalline hydroxyapatite interactions, depending on the pH value of the solution from which the n-cHAp/L-LysHCl and n-cHAp/L-ArgHCl biomimetic composites were crystallised (Figure 1, Figure 2, Figure 3, Figure 4 and Figure 5).

First, X-ray diffraction data confirm that the hydroxyapatite synthesized using our technique [37], which was used to obtain the n-cHAp/L-LysHCl and n-cHAp/L-ArgHCl composites, is nanocrystalline and has magnesium, sodium and fluoride ions in its structure, which was confirmed for these samples by previous X-ray photoelectron spectroscopy (XPS) studies [37].

Second, the results of X-ray diffraction phase analysis of the amino acids crystallized from media with different pH values agree with previously published data on the change in the charge state of L-Lysine and L-Arginine under increased or decreased medium acidity [41,52], which is reflected in the redistribution of the diffraction peak intensities. Thus, the increased intensity of the diffraction reflex (300) of n-cHAp in the presence of L-Arginine in the charged state means the influence of the amino acid on the orientation of the n-cHAp crystals [65]. In turn, orientation of the n-cHAp crystals in the n-cHAp/L-LysHCl biocomposite changes only when L-Lysine is positively charged, above the isoelectric point (pH < 8.7). The ratio of the intensities of the L-Lysine diffraction reflexes (150) and (420) in the n-cHAp/L-LysHCl biocomposite at pH ≤ 5 is similar to the ratio of these peaks for L-Lysine sample in the anionic form (pH ≥ 11.2), indicating the charge compensation that occurs between hydroxyapatite and L-Lysine (Figure 2a).

Analysis of the FTIR spectrum of the synthesised n-cHAp showed (Figure 5a,b curve 1) that this sample is characterised by the presence of low-intensity absorption bands corresponding to the valence and strain vibrations of structurally bonded OH groups at 630 cm^−1^. It should be noted that the intensity of the absorption bands of OH groups in the spectra of the n-cHAp samples (Figure 5) is significantly lower than in the spectrum of the stoichiometric HAp sample [66]. It has been repeatedly shown that isomorphic substitutions in the structure of hydroxyapatite lead to the formation of various defects, including vacancies in the positions of OH groups [67]. Thus, the crystal structure of the n-cHAp samples synthesized in our work is characterized by the substitution of the phosphate ion PO_4_^3−^ by the CO_3_^2−^ group (B-type of substitution). Absorption bands of CO_3_ groups in the spectra of n-cHAp samples were recorded in the range of 1400–1470 cm^−1^. The formation of defects in the n-cHAp crystal lattice leads to a decrease of the intensity of absorption bands for OH groups in the FTIR spectra of the synthesized samples (Figure 5a,b curve 1).

The FTIR spectra of all the biomimetic synthesised composites showed absorption bands that could not be assigned to n-cHAp or the corresponding amino acid (Figure 5a,b curves 2, 3, 4).

The results of the FTIR spectroscopic investigation of the change in the charge state of L-Lysine in the environments with different alkalinity are consistent with the results of X-ray diffraction analysis as evidenced by the redistribution of intensities of modes in the spectra correlated with the side chains, i.e., amide and carboxyl groups, of the amino acid. The assumption of charge compensation between n-cHAp and L-Lysine in the cationic form is also confirmed, as evidenced by the changes in the ratio of intensities of modes attributed to the OH groups of hydroxyapatite and the vibrations of the phosphorus-oxygen tetrahedrons acid (Figure 5a curves 2, 3, 4). It can be concluded that the interaction between n-cHAp and L-Lysine occurs through the formation of chemical bonds. Binding between the PO_4_^3−^ group of n-cHAp and the NH_3_^+^ group of L-Lysine occurs mainly in an acidic (pH ≤ 5) environment, whereas the substitution of OH groups of hydroxyapatite by the carboxyl group of L-Lysine occurs mainly in an alkaline environment. The adsorbed amino acids molecules occupy the Ca and P sites of the HAP surfaces. Thus, L-Lysine changes its charge state with increased or decreased alkalinity of the environment, and n-cHAp participates in the neutralisation reaction with L-Lysine in cationic form. These processes of interaction were previously simulated in [16,41,68].

As for the samples of L-Arginine crystallised from the media with different pH values, small redistributions of the intensities of modes attributed to the vibrations of amide groups are observed depending on the medium acidity (Figure 4b). This observation represents changes in the conformation of L-Arginine molecules in the environments with different acidities [41,65]. The introduction of n-cHAp into the L-Arginine matrix leads to a change in the intensity of absorption bands near 1090 cm^−1^ and 962 cm^−1^, related to the vibrations of phosphorus-oxygen tetrahedrons of hydroxyapatite (Figure 5b). In addition, a redistribution in the intensities of modes attributed to the vibrations of L-Arginine amide groups in the samples of n-cHAp/L-ArgHCl prepared at the increased and decreased alkalinity is observed. The presence of an additional NH_3_^+^ group in L-Arginine hydrochloride and the absence of a COO^−^ side chain led to the mutual orientation of L-Arginine hydrochloride and n-cHAp molecules. Thus, the X-ray diffraction and infrared spectroscopy results confirm that the changes in the molecular composition of biomimetic composites are caused by the electrostatic interaction between the L-ArgHCl molecule and the carbonate-substituted calcium hydroxyapatite.

Thus, the problems of bio-inspired material synthesis dealt with in our work, as well as the establishing of the mechanisms of organic-mineral interaction in biomimetic composites, will enable the achievement of optimal biocompatibility between the native tissue and dental biocomposite, and, as a result, to develop an optimal therapeutic approach for the treatment of dental diseases. 

## 5. Conclusions

Using structural and spectroscopic analysis methods, differences in the surface interaction of nanocrystalline non-stoichiometric carbonate-substituted hydroxyapatite and the polar amino acids L-Lysine hydrochloride and L-Arginine hydrochloride in acidic and alkaline media were established. 

X-ray diffraction data confirm that the hydroxyapatite synthesized using our technique, which was used to create the n-cHAp/L-LysHCl and n-cHAp/L-ArgHCl composites, is nanocrystalline and has magnesium, sodium and fluoride ions in its structure that was confirmed for these samples by our previous XPS studies. The results show that L-Arginine hydrochloride retains its amorphous structure in the presence of hydroxyapatite. Thus, with the formation of n-cHAp/L-LysHCl biocomposites, the directional agglomeration of hydroxyapatite occurs, unlike in n-cHAp/L-ArgHCl biocomposites, for which a homogeneous distribution of hydroxyapatite in the amino acid matrix is observed.

Studies of molecular composition of the samples by Fourier transform infrared spectroscopy under the change in the charge state of L-Lysine in environments with different alkalinity are consistent with the results of X-ray diffraction analysis, as is evidenced by the redistribution of intensities of modes in the spectra correlated with the side chains, i.e., amide and carboxyl groups, of the amino acid. The formation of a biocomposite containing nanocrystalline non-stoichiometric carbonate-substituted hydroxyapatite and L-Lysine occurs through the formation of chemical bonds. Binding between the PO_4_^3−^ group of n-cHAp and the NH_3_^+^ group of L-Lysine occurs mainly in an acidic (pH ≤ 5) environment, whereas the substitution of OH groups of hydroxyapatite by the carboxyl group of L-Lysine occurs mainly in an alkaline environment. The results of the structural and spectroscopic analysis indicate the chemical adsorption of L-Lysine onto the surface of the hydroxyapatite.

Corresponding studies of the molecular structure of biomimetic composites containing L-Arginine showed that the interaction of this amino acid with the carbonate-substituted hydroxyapatite involved mechanisms of molecular orientation observed through changing of vibrational modes correlated with the carbon chain and the guanidine group (CN_3_H_5_^+^) of L-Arginine, and also weakly depends on the pH value of the medium. The X-ray diffraction and infrared spectroscopy results confirm that changes in the molecular composition of n-cHAp/L-ArgHCl biomimetic composites are caused by the electrostatic interaction between the L-ArgHCl molecule and the carbonate-substituted calcium hydroxyapatite.

The revealed mechanisms of interaction of n-cHAp, with a set of physicochemical properties characteristic for the apatite of human tooth enamel, and specific polar amino acids, are crucial for selecting of the formation conditions for biomimetic composites and their integration with natural dental tissue.

## Figures and Tables

**Table 1:** Methods of structural and spectroscopic analysis.

Detection Features	Techniques		
	X-ray Structural Analysis	Synchrotron Fourier Transform Infrared Spectroscopy	Transmission Electron Microscopy
***Identification***	Crystalline structure	Molecular and chemical bonds	Morphology of compounds, surface geometry
***Source for characterisation***	X-rays	Synchrotron radiation in Infrared range of spectrum	Electrons
***Resolution***	Angstrom	cm^−1^	nm
***Description***	Providing information on structures, phases, preferred crystal orientations (texture), and other structural parameters of the dried solution samples	Identify or characterize organic/bio materials through creating a spectrum that shows molecular vibrations	Imagining and characterization of nanoparticles with high spatial resolution

**Table 2:** Active modes in the FTIR absorption spectra of L-LysHCl samples and n-cHAp/L-LysHCl biocomposites, as well as their molecular group assignments.

Active Modes	Frequency of the Absorption Bands in the FTIR Spectra of the Samples, cm^−1^						Ref.: Study Reference Number
	L-LysHCl			n-cHAp/L-LysHCl Composites			
	pH ≤ 5	pH ≥ 7.5	pH ≥ 11.2	pH ≤ 5	pH ≥ 7.5	pH ≥ 11.2	
***δ_as_* NH_3_^+^**	1637 1629 1610	1635 1621 1612	1639 1627 1616	1642 1624 1612	1645 1628 1610	1651 1622 1609	[41,46,47,51,52]
***δ* CH_32_^+^**	1470	1472	1468	1469 1459	1465 1460	1465 1459	[41,46,47,51]
***v_s_* COO^−^**	1415	1416	1414	1413	1417	1415	[41,46,53]
***ω* C*γ, τ* C*δ, τ* C*ε***	1348	1349	1348	1347	1349	1348	[41,46,47]
***ρ*_as_ NH_3_^+^, *τ* C*β,* C*β-*C*α-*H*α, ω* C*δ***	1220	1222	-	-	1222	1222	[41,46,47]
***ρ_as_* NH_3_^+^, *ρ* Cε**	1182	1186	-	-	1185	1186	[41,46,47]
***ρ_as_* NH_3_^+^,** **C-Cα-Hα**	1148	1145	1148	1161	1155	1155	[41,46,47]
**PO_4_^3−^, *v*_3_**	- -	- -	- -	1089 1027	1091 1024	1099 1024	[37,39,54,55]
**PO_4_^3−^,*v*_1_**	-	-	-	970	966	974	[37,39,54,55]
***v* C-C, *v* C-N** ***ω* C*γ, τ* C*δ, τ* C*ε,***	1002 975 937 911	1003 978 935 907	1007 979 937 912	1003 961 935 912	1003 961 935 908	1003 978 935 905	[41,46,47,51,53]
**τ,ω NH**	805	802	-	-	803	803	[41,46]
***ρ* CH_2_**	785 764	782 764	788 -	787 -	782 -	782 764	
***ρ* CH_2_, *v* C-C,** ***δ* COO**	704	-	708	702	704	-	[47,52,56]
**OH (n-cHap)**	-	-	-	632	630	631	[37,39,54,55]
**PO_4_^3−^,*v_4_***	-	-	-	602 558	602 559	601 560	[37,39,54,55]
***ρ,* ω, *δ,*** **COO^−^**	560 551	562 555	560 553	554	569 550	570 547	[51,57]
**NH_3_^+^_torsion_**	477	477–480	460–480	464–478	473	474	[57]

**Table 3:** Active modes in the FTIR absorption spectra of L-ArgHCl samples and n-cHAp/L-ArgHCl biocomposites, and their molecular group assignments.

Active Modes	Frequency of the Absorption Bands in the FTIR Spectra of the Samples, cm^−1^						Ref.: Study Reference Number
	L-ArgHCl			Composites n-cHAp/L-ArgHCl			
	pH ≤ 5	pH ≥ 7.5	pH ≥ 11.2	pH ≤ 5	pH ≥ 7.5	pH ≥ 11.2	
***v*_s_ CN_3_H_5_^+^,** ***v*_as_ NH_3_^+^**	1678 1666	1678 1669	1675 1667	1680 1667	1678 1669	1680 1669	[41,56,58,59]
***v*_as_ CN_3_H_5_^+^,** ***v*_s_ NH_3_^+^**	1629 1620	1630 1621	1631 1621	1632 1620	1630 1617	1632 1619	[41,56,58,59]
***v*_s_ NH_3_^+^**	1543	1543	1543	1543	1541	1541	[41,56,58,59]
***δ* CH_2_**	1467 1449	1467 1449	1467 1449	1466 1454	1472 1456	1469 1449	[41,56,58,59]
***v*_s_ COO^−^**	1401	1401	1401	1402	1398	1400	[41,56,58,59]
**N-Cα-Hα,** **Cβ-Cα-Hα**	1356	1362	1356	1360	1361	1361	[41,56,58,59]
**Cβ twisting,** **Cγ rocking,** **Cβ-Cα-Hα**	1307	1307	1307	1307	1309	1308	[41,56,58,59]
***δ, ρ* NH_3_^+^**	1204 1188 1167	1205 1185 1168	1207 1185 1168	1207 1193 1153	1212 1198 1167	1208 1195 1169	[41,56,58,59]
**v_as_ CN_3_H_5_^+^**	1127	1123	1120	-	-	-	[41,56,58,59]
***v*_3_ PO_4_^3−^**	- -	- -	- -	1089 1027	1091 1024	1099 1024	[37,39,54,55]
***ρ* CH_2_**	-	985	983	-	-	-	[41,56,58,59]
***v* C-C**	885	886	890	-	-	-	[41,56,58,59]
***v*_1_ PO_4_^3−^**	-	-	-	970	966	974	[37,39,54,55]
***ω*, *δ* COO^−^**	630 593	632 594	639 588	573	573	574	[46]
**OH**	-	-	-	629	631	632	[37,39,54,55]
***v_4_* PO_4_^3−^**	-	-	-	600 559	600 558	599 558	[37,39,54,55]
**τ NH_3_^+^**	526	525	524	530	526	524	[41,56,58,59]

## Data Availability

The data that support the findings of this study are available from the corresponding author upon reasonable request.
